# Single-Cell Heterogeneity of Cutaneous T-Cell Lymphomas Revealed Using RNA-Seq Technologies

**DOI:** 10.3390/cancers12082129

**Published:** 2020-07-31

**Authors:** Karolina Rassek, Katarzyna Iżykowska

**Affiliations:** Institute of Human Genetics, Polish Academy of Sciences, 60-479 Poznań, Poland; katarzyna.izykowska@igcz.poznan.pl

**Keywords:** Sézary syndrome, mycosis fungoides, cutaneous T-cell lymphomas, single-cell heterogeneity, RNA-sequencing

## Abstract

Cutaneous T-cell lymphomas (CTCLs) represent a large, heterogeneous group of non-Hodgkin lymphomas that primarily affect the skin. Among multiple CTCL variants, the most prevalent types are mycosis fungoides (MF) and Sézary syndrome (SS). In the past decade, the molecular genetics of CTCL have been the target of intense study, increasing the knowledge of CTCL genomic alterations, discovering novel biomarkers, and potential targets for patient-specific therapy. However, the detailed pathogenesis of CTCL development still needs to be discovered. This review aims to summarize the novel insights into molecular heterogeneity of malignant cells using high-throughput technologies, such as RNA sequencing and single-cell RNA sequencing, which might be useful to identify tumour-specific molecular signatures and, therefore, offer guidance for therapy, diagnosis, and prognosis of CTCL.

## 1. Introduction

Cutaneous T-cell lymphomas (CTCLs) are a large, heterogeneous group of lymphoproliferative hyperplasias derived from mature skin-homing T lymphocytes, with a different stage of malignancy. Sézary Syndrome (SS) and Mycosis fungoides (MF) constitute about 65–80% of all CTCLs cases [1]. MF is the most prevalent form of CTCL, constituting approximately 50% of all lymphomas arising primarily in the skin, with an incidence rate of six to seven cases per million persons. Early-stage MF has an excellent prognosis, and 90% of patients do not progress to the tumour stage [2,3]. The incidence of SS, an aggressive and leukemic variant of CTCL, is 0.1–0.3 cases per million persons, accounting for only 2.5% of all CTCLs [4]. MF affects African Americans more often than Caucasians, while the incidence rate of SS is higher in Caucasians than in African Americans. However, the 2:1 male-to-female ratio is the same in both lymphomas [3,4]. Both SS and MF were shown to be closely related; the clinical features of the late-stage MF might resemble those of SS. Moreover, both diseases are characterized by similar genetic profiles and by great diversity in gene expression, mutations, and chromosomal aberrations [5]. Despite much study, the driver mutations of CTCLs are still unknown [2]. Moreover, due to the lack of specific markers and resemblance to different dermatologic conditions, such as chronic actinic dermatitis, psoriasis, idiopathic erythroderma or chronic eczematous dermatitis, the diagnosis of CTCLs is very challenging, with limited treatment options available [6,7,8]. Recent studies on genotyping and immunophenotyping of CTCLs indicate that, although arising as an expansion of mature helper memory T-cells, the population of cells derived from each patient shows some level of heterogeneity by forming complex aberrant clonal hierarchies and subclones [9,10,11,12]. Determining intratumoral heterogeneity is challenging, but is crucial for understanding tumour pathogenesis and evolution, and might have important implications for the diagnosis and treatment [13]. In this review, we highlight recent insights into the genomic heterogeneity of CTCLs through the application of novel RNA sequencing approaches.

## 2. Clinical and Immunological Features of CTCLs

Classic Alibert–Bazin MF is characterized by the progressive appearance of plaques, patches, and, ultimately, in the case of some patients, tumours [3]. Malignant lymphocytes of MF resemble those of the *α*/*β* memory T-helper phenotype (T-cell receptor [TCR]ß^+^, TCRγ^−^, cluster of differentiation CD3^+^, CD4^+^, CD5^+^, CD8^−^, CD45RO^+^, T-cell intracellular antigen [TIA]-1^−^). In most conventional cases of MF, cells do not express cytotoxic markers, such as TIA-1, granzyme B, and perforin. However, sometimes, these cells can exhibit a T-cytotoxic phenotype (TCRß^+^, TCRγ^−^, CD3^+^, CD4^−^, CD5^+^, CD8^+^, TIA-1^+^ or TCRß^−^, TCRγ^+^, CD3^+^, CD4^−^, CD5^+^, CD8^+^/^−^, TIA-1^+^). In advanced stages of MF, CD4^+^/CD8^+^ or CD4^-^/CD8^-^ phenotypes can be observed [3].

SS is typically characterized by erythroderma, lymphadenopathy, and severe pruritus. Neoplastic T lymphocytes (Sézary cells) present in skin, lymph nodes, and peripheral blood express the CD3^+^CD4^+^CD8^−^ phenotype. Expression of CD3, CD4, CD45RO, and CCR4 indicates a mature memory T-cell phenotype, and expression of CCR7, L-selectin, and CD27, a central memory T-cells phenotype of malignant cells. Sézary cells also express T-regulatory profile (CD25 and FOX-P3) phenotypes, which result in suppression of the immune response [4]. 

Both MF and SS lymphocytes can express a T-helper type 2 phenotype, characterized by inreased IL-4, IL-5, IL-10 and IL-13 production [14]. In early MF Th1 phenotype could be detected, but it switches to Th2 as this phenotype creates more beneficial microenvironmet for tumor growth. The role of Th17 and Th22 cells in the pathogenesis of CTCL was also investigated and it was shown that IL-22 is higly expressed in lesional skin of CTCL, in contrast to low expression of Il-17. 

## 3. High-Throughput RNA Sequencing Techniques

High-throughput technologies, such as RNA sequencing (RNA-seq), have become irreplaceable tools for transcriptional analysis of differential gene expression. By sequencing a huge number of cells from one sample, it is now possible to investigate aspects of RNA biology, such as its structure, interactions, and pathways of translation or transcription [15]. Because of unbiased analysis of the entire transcriptome, RNA sequencing enables us to identify previously undescribed transcripts, such as lncRNAs, gene isoforms, or pathways of gene expression regulated by enhancer RNAs. Another advantage of the RNA-seq method is the ability to identify non-human transcripts, for example, those of viral origin, that can confirm or exclude a potential infectious aetiology of human diseases [16,17]. 

**Figure 1:**
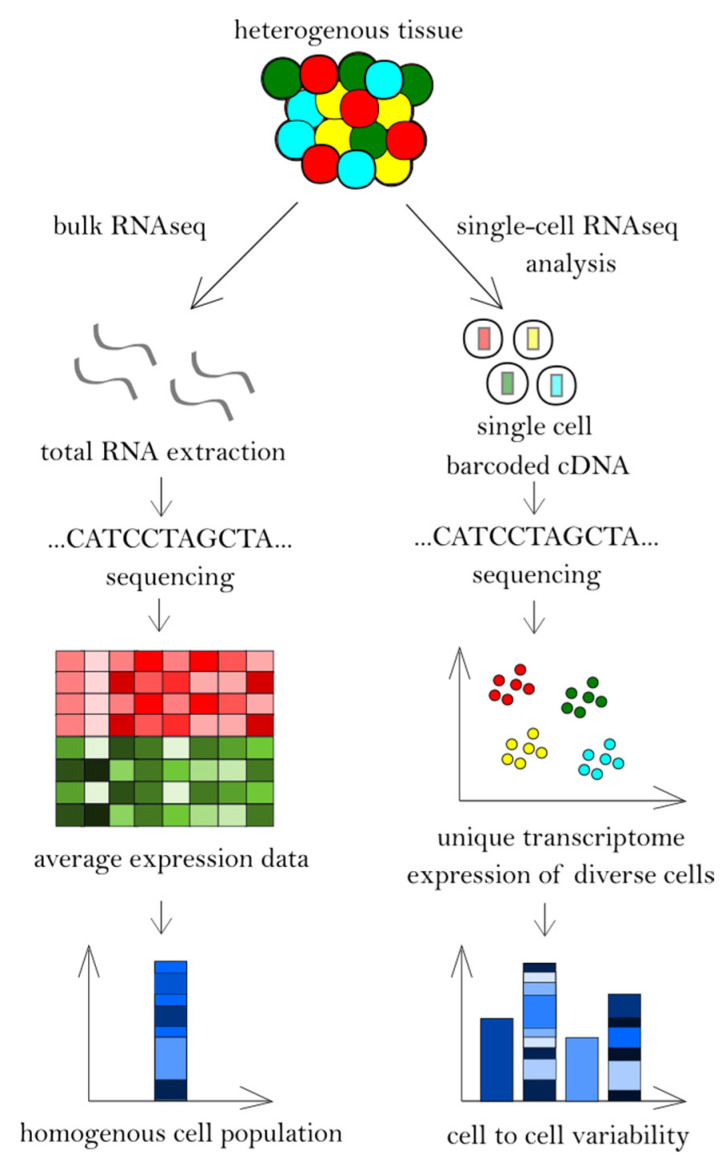
Bulk RNA sequencing and single-cell RNA sequencing workflow.

Single-cell RNA sequencing, a recent development of RNA-seq, is a revolutionary tool with several distinct advantages over bulk RNA-seq, such as investigation of expression patterns of individual cells. By using scRNA-seq, it is now possible to track cell lineages during differentiation or examine rare cell populations, which could not be detected using bulk RNA-seq [18,19]. Many scRNA-seq protocols and approaches have been introduced during method development. However, all of them follow the same basic steps. Common principles required for the generation of scRNA-seq libraries include the isolation of cells from each other, cell lysis, reverse-transcription into the first-strand cDNA, and cDNA amplification [20]. Although experimental methods are increasingly developing, there are still some important drawbacks of scRNA-seq that should be considered. Because of the low amount of material, there is a low mRNA capture efficiency and a high dropout rate. Therefore, an efficient cell lysis strategy is needed. Additionally, compared to bulk RNA-seq, scRNA-seq produces more variable and nosier data, which pose challenges for the computational analysis of the results. Although some tools have been designed and commercial companies (e.g., 10× Genomics and Illumina) have provided software to handle raw data files, this area requires further improvement (Table 1), (Figure 1) [19,21].

## 4. RNA Seq Analysis of CTCL Patients

Litvinov et al. were the first to use TruSeq targeted RNA gene expression analysis to study formalin-fixed and paraffin-embedded (FFPE) samples from a cohort of CTCL patients and benign inflammatory dermatoses [22]. The comparison of gene expression using clustering analysis revealed a significant cross-classification between CTCL and benign samples, and highlighted a significant degree of heterogeneity with respect to gene expression changes within different CTCL samples and even within the same patient, where samples taken at different times did not cluster together. The analysis of over 280 highly studied biomarkers and candidate genes for CTCL pathogenesis confirmed several important gene expression changes that, in combination with other techniques, have diagnostic and prognostic potential. The authors especially underlined the upregulation of the TOX, FYB, LEF1, CCR4, ITK, EED, POU2AF, IL-26, STAT5, BLK, and GTSF1 genes and downregulation of the PSORS1C2 gene in CTCL patients compared to controls (benign inflammatory dermatoses). The overexpression of the thymocyte selection-associated high-mobility group box (TOX) gene was especially highlighted, as TOX was previously reported to be upregulated in MF and SS and correlated with increased risk of disease progression and poor prognosis [23,24]. In this study, the statistical analysis of gene expression between early and advanced stages of CTCLs revealed upregulation of the TOX, FYB, and GTSF1 genes and downregulation of the LTB4 gene in advanced stages of the disease, consistent with previous studies that identified those genes as molecular markers of progression [25]. Moreover, it was shown that the TOX, FYB, and CCR4 genes are upregulated in stage I patients that were at risk of cancer progression. The study also revealed overexpression of STAT5 in CTCL samples, which was previously shown as a driver of expression of oncogenic BIC/miR-155 in cancer and promoter of the proliferation of malignant T-cells [26]. Moreover, upregulation of various inflammation mediating genes, such as CD70, STAT signalling genes, LTA, NFKB1, NFKB2, IL-15, and other inflammatory cytokines was observed in CTCL samples compared to controls. The analysis of several selected genes with respect to the clinical stage of the disease enabled the authors to identify upregulation of genes connected with poor prognosis or inflammation. The CD30, GNLY, CD70, and GTSF1 genes were expressed at later stages of the disease, while in early stages, BCL7A (a favourable prognosis gene) was expressed. The bioinformatics follow up of the Litvinov et al. study was conducted by another group of Lefrancois et al. TruSeq gene expression patterns in older (≤2008) vs. more recent (≥2009) FFPE samples were analyzed in order to examine if previous clustering and gene expression patterns can be confirmed when analyzed based on the year of biopsy [27]. Both analyses showed nearly identical trends and findings. In addition, Lefrancois et al. validated known upregulated in CTCL targets such as STAT signaling genes, and inflammatory interleukins and identified novel differentially expressed genes that were not statistical significant in Litvinov et al. study, including upregulated: BCL11A, SELL, IRF1, MAD1, CASP1, BIRC5 and MAX and downregulated MDM4, SERPINB3 and TBS4 genes. 

To investigate the mutational landscape of SS genomes and possible fusion transcripts, Prasad et al. analyzed SS samples using whole-exome sequencing and RNA-seq [28]. Fusion transcripts that are expressed on the RNA level as a result of genomic rearrangements are often involved in malignant transformation as they might result in disruption of tumour suppressor genes or activation of oncogenes. In ten SS patients analyzed, 86 potential fusion transcripts were detected. Among them, TYK2-UPF1, COL25A1-NFKB2, FASN-SGMS1, SGMS1-ZEB1, SPATA21-RASA2, PITRM1-HK1, and BCR-NDUFAF6 were validated and discussed to have a potential role in pathogenesis due to the involvement of a fusion partner in signaling pathways, T-cell differentiation, transcriptional regulation, or proliferation. 

Fusion transcripts were also identified in the SS patients studied by Iżykowska et al. [29] and Wang et al. [30]. Iżykowska et al., using whole genome sequencing and RNA-seq technology, analyzed 9 SS patients and SS derived cell line, SeAx. Many copy number variations and rearrangements were detected, fifteen rearrangements resulted in the expression of new fusion transcripts [29], with only one (TFG-GPR128) reported before [31]. Five of the detected transcripts resulted in ectopic expression of fragments of genes not present in normal T- cells (BAIAP2, CPN2, GPR128, CAPN12 and FIGLA) and nine of the transcripts were in frame (EHD1-CAPN12, TMEM66-BAIAP2, MBD4-PTPRC, PTPRC-CPN2, MYB-MBNL1, TFGGPR128, MAP4K3-FIGLA, DCP1A-CCL27, MBNL1-KIAA2018). Wang et al. investigated CD4+ T-cells from peripheral blood of 37 advanced stage SS patients [30]. Among 41 in-frame fusions, 29 were validated, and one of them, CD28-CTLA4, was previously detected in CTCLs. CTLA4–CD28 gene fusion was detected in several types of lymphoma [32] and thus could provide a target for potential immunotherapy. A case study was even conducted where a SS patient was treated with a CTLA4 inhibitor [33]. Recently, it was proposed that, in cancer immunotherapy, the CD28 agonists could be used together with anti-PD1 antibodies to increase the effectiveness of therapies targeting PD1, which are also tested in terms of MF/SS [34,35]. Moreover, using RNA-seq, Wang et al. [30] was able to distinguish 345 upregulated transcripts. Several CD molecules and chemokines required for T-cell development and function, as well as interleukins and interleukin receptors, were upregulated (IL32, IL2RG, CD3G, CD27, CCR4, and CCR8). High expression of IL2RG, which encodes IL2 receptor common gamma chain, was detected in all examined patients. The most upregulated chemokine in all but one patient was IL32, a proinflammatory cytokine important in T-cell communication, tumorigenesis, and autoimmune diseases. Previous studies have suggested that IL32 might be involved in an autocrine signalling loop stimulating the growth of Sézary cells and that high expression of IL32 in MF patients is correlated with disease activity [36].

## 5. Identification of *Long Non*-*Coding* RNAs in CTCL

The development of high-throughput sequencing technologies has enabled the detection and classification of cancer-associated non-coding RNA. Long non-coding RNAs (lncRNAs) are classified as more than 200 nt long transcripts, which lack protein-coding potential. It has been shown that lncRNAs are involved in many cellular processes, such as chromosome structure modulation, transcription, splicing, and post-translational modifications [37]. In recent years, lncRNAs dysregulation has been linked to the pathogenesis of some disorders, such as cardiovascular diseases, metabolic disorders, and cancer [38,39,40]. Moreover, it has been suggested that lncRNAs can serve as potential diagnostic and prognostic markers [41,42] or targets of drug treatment in some cancers [43]. Therefore, the reliable identification of lncRNAs might be critical for understanding the molecular pathogenesis of CTCLs. Because the RNA-seq technique is more sensitive to detecting less-abundant transcripts and identifying novel splicing isoforms, it is a technique of choice to study gene expression signatures specific to tissues or cell types [44]. 

To obtain a pure population and minimize the detection of less relevant differences in mRNA expression, Lee et al. compared Sézary cells (SCs) to patient-matched polyclonal CD4+ T-cells from three individuals [45]. In this study, the role of lncRNAs in SS and MF was investigated for the first time. The authors identified 21 annotated SC-associated lncRNAs differentially expressed in SS cells, and the presence of them in the majority of 24 examined MF tumours was confirmed. Among them, there were 13 previously unreported Sézary cell-associated transcripts (SeCATs) with differential expression, 12 with highly conserved regions that are predicted to be noncoding and have potential functional importance. Furthermore, Lee et al. performed the analysis of protein-coding genes and found that 525 were commonly upregulated and 519 were downregulated. Within 1044 genes, they detected upregulation of: TNFSF11 (RANKL), PTHLH, EPHA4, ZNF331, DDX41, KCNN4, ITGB1, CNIH4, and CD52 and downregulation of APBA2, STAT4, NEDD4L, MXI1, TGFBR2, BCL2L11, SATB1, SP140, and RPS2, as reported in previous studies [46,47,48]. Moreover, the study revealed an increased expression of several genes that encode transmembrane (TMEM) proteins in all three SS patients: ACVR2A, ADAM8, ANK1, APP, CD4, CD59, EMP3, EPHA4, GPR68, KCNN4, PDCD1, PSEN1, SIGIRR, and TNFRSF1B. The authors noted that presenilin-1 is a part of the y-secretase complex, important in the oncogenic pathway in T-cell acute lymphoblastic leukaemia [49], that KCNN4 seems to be responsible for T-cell activation and proliferation [50], and that PCDCD1 is considered to be important in T-cell function and contributes to the prevention of autoimmune diseases [51]. Given their accessibility to therapeutic antibodies, TMEM proteins seem to be attractive targets for future studies.

## 6. Single-Cell RNA Seq in CTCL Studies

### 6.1. The Population of Malignant Sézary Cells can be Divided into Distinct Subpopulations

Surface antigens signatures are important for establishing cells in different conditions or unravelling molecular changes in cells, and therefore achieving a better understanding of the disease [52]. Furthermore, the identification of populations of cells exhibiting different gene signatures is crucial for effective clinical treatment. Researchers in the following studies used scRNA-seq to analyze blood samples and skin biopsies of CTCLs patients to identify potential tumour-specific molecular signatures. 

Buus et al. investigated peripheral blood mononuclear cells from seven patients diagnosed with SS [10]. Using the T-distributed Stochastic Neighbor Embedding (t-SNE) visualization algorithm of single-cell expression of multiple markers at the same time, they were able to divide the population of malignant cells into subpopulations exhibiting distinct combinations of surface markers. The analysis of those populations showed that not all patients had similar heterogeneity of all examined markers and all markers showed differential expression within at least one patient. Only cutaneous lymphocyte-associated antigen (CLA) had bimodal expression in the malignant populations in all patients. Heterogeneous surface phenotypes were correlated to distinct mRNA transcript profiles within the malignant population studied in six SS patients. T-cell relevant genes were analyzed and, similarly to the expression of surface markers, mRNA transcripts also divided malignant cells into subpopulations. Among 110 T-cell related genes examined, only five (S100A4, S100A10, IL7R, CCR7, and CXCR4) were highly expressed by most of the malignant cells, and two of them (cancer-related genes S100A4 and S100A10) were ubiquitously expressed in all populations. A good correlation between the mRNA and protein expression at the single-cell level for the investigated genes (SELL, IL7R, CCR7, and CD4) encoding proteins was observed.

A study published by Borcherding et al. was based on single-cell RNA and T-cell receptor sequencing and comparison of pooled SS cells to normal CD4+ controls [12]. In an analyzed SS patient, 12 clusters were distinguished based on mRNA expression, and each cluster was defined by expression of five to seven top genes. Clusters were most closely associated with normal versus malignant cells, as six of them were constituted of normal CD4+ T-cells and five of malignant SS cells. The gene expression analysis of known marker genes showed that the majority of cells of both normal and malignant origin correlated with CD4+ central memory T-cells. The malignant population was clonal and exhibited an increase in expression of CD70 and a decrease in CD26 expression, also identified in multiple inflammatory diseases [53]. Moreover, malignant cells had increased CD5 expression and maintained a CD7 expression, which is usually lost in the case of CTCLs patients [54]. Normal and malignant cells were further analyzed in terms of differential gene expression, and several marker genes were identified; most of these had been previously described, but two were newly identified: SAMSN1 and TSPAN2. 

Gaydosik et al. investigated skin samples from five CTCL patients and four healthy controls [11]. Cells were grouped according to expression profiles and, unlike in healthy samples where there was an overlap between cells, no overlap was detected between cells from both tumour and healthy samples or between tumour samples themselves. Based on the comparison of the transcriptomes of each lymphocyte subset from tumours and from four skin control samples, at least one cluster unique for each sample was identified. Those clusters were identified based on the expression of unique genes that were selected by differential expression analysis. Moreover, those unique clusters expressed TOX, a marker of malignant lymphocytes, and a significant although heterogeneous over-expression of genes associated with tumour cell proliferation, tumorigenesis, and resistance to apoptosis. In addition, gene expression analysis allowed the authors to identify highly proliferating lymphocytes and those clusters had 17-gene expression signature common to all tumours: ACTG1, ANP32B, ATP5C1, DUT, HMGN1, HN1, NPM1, NUSAP1, PCNA, PPA1, PPIA, PSMB2, RAN, RANBP1, SET, SMC4, and STMN1. Three of those genes, PCNA, ATP5C1, and NUSAP1, were confirmed to be co-expressed with TOX in tumour samples, but not in normal skin and atopic dermatitis, and as a result, they have a potential to be a diagnostic marker in CTCL. 

### 6.2. Heterogeneity of Malignant Population

The main challenge in cancer diagnosis and effective treatment is tumour heterogeneity. Single-cell RNA sequencing has enabled the gathering of molecular profiles for thousands of individual cells, thus enabling the quantitative characterization of cell heterogeneity. Identifying the origins of cellular heterogeneity and understanding how individual cells process information and respond to signals has now become a central challenge of medicine.

SS malignant cells are heterogeneous in terms of surface protein expression and mRNA profile. Buus et al., using single-cell methods, showed that SS cells have a heterogeneous expression of different T-cell markers that divide the population of malignant cells into subpopulations difficult to classified based on conventional T-cell classification [10]. Previously, it has been proposed that SS cells originate from central memory T-cells because of the expression of CD45RO, CD62L, and CD197. However, using flow cytometry, Buus et al. revealed that many malignant cells from SS patients expressed CD45RA, which is a marker of naïve T lymphocytes or stem-cell memory lymphocytes. Moreover, mRNA sequencing of 110 T-cell related genes revealed heterogeneous expression patterns between malignant T-cells. Surprisingly, even genes that have been considered to be classical biomarkers of CTCLs (ILR7, CCR7, and CXCR4) were heterogeneously expressed within some patients. 

Borcherding et al. investigated the heterogeneity of the SS cells at the single-cell level and separated the malignant population into five clusters having distinct transcriptional states [12]. Differences in transcription factors expression between malignant clusters were analyzed, and a clonal evolution was predicted to start from FOXP3 positive cells to both GATA3A+ and IKZF2+. Moreover, immune phenotypes were investigated, and cells were analyzed for markers of skin-homing T-cells, central memory cells, and Tregs. Low expression of FUT7 and consistent expression of skin-homing markers (CCR4, SELPLG, and ITGB1) and central memory markers (CD28, CCr7, and SELL) have been noticed. Low expression of CD25, a Treg marker, was noticed in all clusters, but in one cluster there was a distinct population having a Treg cell-like phenotype. Functional heterogeneity between malignant cells was also confirmed based on analysis of different T-cell related gene sets and pathways. 

Gaydosik et al., based on analysis of transcriptional profiles of T lymphocytes, identified large inter- and intratumour heterogeneity in advanced CTCLs skin samples [11]. A cluster analysis showed that each patient has a unique cluster characterized by expression of certain genes: RDH10, CXCL13, SCG2 (CTCL-2), FGR, IGFBP2/P6, NEFM (CTCL-5), ANO1, TNP1, CES4A, ZDHHC14 (CTCL-6), LGALS7, SERPINB3/B4, SPR2A (CTCL-8), NTRK2, and TMPRSS3 (CTCL-12). Furthermore, the expression signature of tumour-specific clusters showed activation of specific tumour-associated pathways involved in tumour cell survival, proliferation, and metastasis. Heterogeneity was also detected in the microenvironment of tumour cells, especially in the population of tumour-infiltrating CD8+ lymphocytes (TILs), which are responsible for killing cancer cells, but they are often incapable of mounting an efficient anti-tumour action. The molecular signature of those cells was based on analysis of effector molecules, checkpoint receptor inhibitors, and Treg markers, and the analysis revealed heterogeneity in both effector and exhaustion programs across patients.

## 7. Dysregulated Signalling Pathways Revealed in CTCL Patients by RNA Sequencing

**Figure 2:**
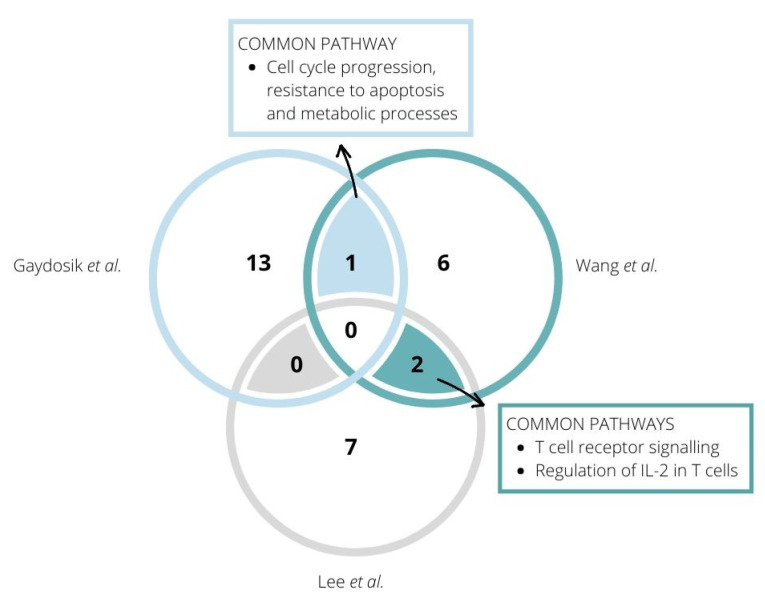
Unique and shared deregulated signaling pathways in Cutaneous T-cell lymphoma (CTCL) cells identified by high-throughput sequencing in the studies by Gaydosik et al., Wang et al. and Lee et al.

High-throughput technologies have also become an important tool for identifying deregulations of specific signalling pathways that might be associated with disease progression and are crucial for understanding and diagnosis of haematological malignancies. So far, the alteration of several pathways has been shown to be involved in the pathogenesis of CTCL, including JAK/STAT signalling, the NFκB signalling pathway, T-cell signalling pathways, TCR associated enzymes, Th2 differentiation, epigenetic regulation, cell survival, and cell cycle checkpoint [30,55,56,57,58]. Consistent with previous reports, the following studies demonstrated a variety of affected pathways in the case of CTCLs. Several pathways, connected with T-cell receptor signalling, IL-2 mediated signalling, and cell cycle progression, were detected in more than one study (Figure 2).

Lee et al.—using genes differentially expressed in three patients—demonstrated the deregulation of several signal transduction pathways in SS cells, including PI3K/Akt, TGFB, NF-kB, and T-cell receptor signalling [45]. Wang et al. performed a gene enrichment analysis that revealed alterations in pathways connected with cell cycle control, regulation of the immune system, TCR signalling, chemokine signalling, and MYC transcriptional activation [30]. 

Gaydosik et al. identified activation of many tumour-associated signalling pathways that are unique to each tumour: activation of eIF2, eIF4 mTOR signalling, NK-cell signalling, and virus entry via endocytic pathways [11]. Moreover, the expression of genes involved in tumour cell survival, proliferation, and metastasis (some common to glioma and non-small cell lung cancer) was identified. In addition, the inactivation of granzyme M—promoting tumour cell transformation, migration and drug resistance and related to skin inflammation and skin barrier dysfunction—was detected, as well as pathways associated with epithelial-mesenchymal-transition. Furthermore, the study reported the activation of pathways common to all proliferating T lymphocytes in each tumour, such as cell cycle progression, resistance to apoptosis, and metabolic processes.

Borcherding et al. found significant differences between malignant clusters in T-cell-related gene sets [12]. This study reported that one cluster, along with the previously noted increase in Tcm and skin-homing gene markers, was significantly enriched for gene signatures of type II interferon signalling, terminal differentiation, and cytolytic activity. Another alteration in gene set enrichment included high levels of hypoxia in another cluster. Contradictory to other clusters, in one cluster, the enrichment of anti-inflammatory and Treg markers was observed.

## 8. Role of Infectious Agents in Disease Onset and Progression

Many studies have investigated potential infectious involvement in triggering or promoting CTCLs. Unfortunately, the results of many of these studies are inconsistent. While a number of studies failed to show any association with pathogenic organisms, studies concluding a definite infectious role in CTCL aetiology have also been reported [59]. Several researchers examined an association with Chlamydophila pneumonia [60,61] and Borrelia burgdorferi [62,63]. However, most of the studies of possible bacterial pathogenesis have focused on investigating the role of Staphylococcus aureus [64,65]. Recently, a potential link between CTCL activity and antibiotic treatment of Staphylococcus aureus has been suggested [66]. Aggressive antibiotic treatment was associated with decreased proliferation of malignant T-cells and inhibition of the disease activity in lesional skin colonized by this pathogen, therefore providing a justification for treatment of Staphylococcus aureus in CTCL patients with severe disease. Attempts have also been made to evaluate the involvement of viral pathogens, such as human T-lymphotropic virus-1 (HTLV-1) [67,68,69,70,71,72,73,74], HTLV-2 [69,75], human immunodeficiency virus (HIV) [76,77,78], Epstein–Barr virus (EBV) [79,80,81,82], human herpesvirus (HHV) 6, 7, and 8 [83,84,85,86], polyomaviruses [87,88], and hepatitis C virus (HCV) [89]. Study of the HTLV-1 virus became especially controversial, as some of the researchers were convinced that it is a crucial factor in the pathogenesis of CTCL [67,68,69], while others indicated that MF and SS are not associated with HTLV-1 infection [70,71,72,73,74]. The study by Netchiporouk et al. focused on highlighting the differences between classic MF/SS an HTLV-1 driven disease [90]. It was shown that although both of the diseases on the molecular level show similar gene expression patterns, there are many differences between them. Classic MF/SS cells are mostly aneuploid, characterized by multiple chromosomal changes and large number of alterations, including mutation of TP53 and strong expression of GTSF1 negative prognostic marker. HTLV-1+ leukemia cells are mostly diploid, with only a minimal number of chromosomal aberrations and structural alterations. HTLV-1+ cells are also characterized by wild type TP53 and weak expression of GTSF1. Moreover, it was shown that while classic MF/SS cells are sensitive to HDAC inhibitor treatment, HTLV-1+ cells are relatively resistant to it. Therefore, authors indicate that HTLV-1 virus is likely not involved in the pathogenesis of CTCLs as it drives a different pathway of lymphomagensis. However, the hypothesis of a common exposure to some infectious agent supports several studies reporting CTCL occurrence in married couples [91], families [92], or even non-blood family members [93].

While searching for the activation of cancer-associated pathways in CTCLs, Gaydosik et al. detected the upregulation of several genes involved with virus entry via endocytic pathways [11]. However, Litvinov et al. were unable to detect HTLV-1 transcript in all but one patient from the endemic area [22]. Lee et al. also failed to reveal the presence of viral transcripts of HTLV-1 or other human viruses in Sézary cells [45]. However, it is possible that these transcripts escaped detection by bulk RNA-seq (but not in the more sensitive single-cell RNA seq method).

## 9. Clinical Significance of Novel Single-Cell RNA Sequencing Technologies

Single-cell techniques open new possibilities that can be used in the clinic, including dealing with drug resistance, designing individual and targeted treatments, and monitoring disease progression. Identification of patient-specific gene expression of malignant cells might be used in personalized therapy [11]. The analysis of unique malignant cells for each patient cluster creates a possibility for accurate therapy focused on specific pathways. On the other hand, signatures common to all tumours, such as TOX gene expression or the expression of certain genes identifying actively-proliferating lymphocytes, could be used for the diagnosis and monitoring of treatment. Importantly, scRNA-seq allows for the study of cells in the cancer microenvironment, such as TILs [11]. Understanding the heterogeneity of co-inhibitory receptor expression might be essential for efficient immunotherapy based on targeting the receptors and enhancing TILs antitumor response. The rapid and accurate diagnosis of the type and stage of the CTCLs is challenging. Borcherding et al. analyzed data from 152 CTCL patients at different stages of the disease using the same algorithm used for the differentiation of unique transcriptional states in malignant population during RNA-seq analysis [12]. Based on this analysis, genes with high expression predictive of early disease (FOXP3 and PTPN6) and late-stage disease (TGFB1, CD7, and SUZ12) were identified, which could be valuable for future diagnostic purposes. Another huge challenge is the resistance of cancer cells to drugs, which is observed beyond the treatment of CTCLs. Only 30% of CTCL patients respond to treatment with HDACi due to HDACi resistance. A recent study showed that there is a molecular explanation for it and it is associated with highly acetylated elements that may drive the high expression of genes promoting disease progression [94]. Using HDACi and single-cell technologies, Buus et al. identified a subpopulation of SS cells that were resistant to treatment [10]. Those resistant cells were classified into the same subpopulations based on the surface markers. The consequences of cells escaping treatment are known to be serious, as they could lead to a relapse of even more aggressive diseases. This knowledge might permit the application of multiple treatments targeting different malignant populations.

## 10. Conclusions

High-throughput technologies provided an unprecedented view into the genomic heterogeneity of CTCLs, allowing a deeper understanding of the pathogenesis and molecular changes of CTCLs. While RNA-seq analysis showed a huge heterogeneity between patients, single-cell RNA-seq technology revealed high intratumor heterogeneity and divided malignant population within a single patient into distinct clusters. Moreover, RNA sequencing allowed the researchers to perform the transcriptome analysis not only in the fresh blood samples but also in FFPE samples, revealing many novel molecular changes, differentially expressed genes and fusion transcripts potentially involved in malignant transformation. The investigation of lncRNAs in CTCL patients uncovered previously unidentified Sézary-associated transcripts, while multiple protein coding and inflammation-mediated genes have been proposed as markers of CTCL progression or diagnosis. The overexpression of the TOX gene was highlighted, as it is considered to be correlated with an increased risk of disease progression and poor prognosis. In addition, multiple dysregulated signaling pathways were identified; however, only a few were common to more than one study. The obtained results also demonstrated a most unlikely involvement of HTLV-1 virus in CTCL pathogenesis.

As the treatment of CTCL patients remains challenging, it is hoped that personalized medicine will enable patient-specific pathways to be targeted during therapy. RNA sequencing technologies, especially those based on the single-cell analysis, can, therefore, help to advance our understanding of the disease and improve treatment.

## Figures and Tables

**Table 1:** Comparison of RNA sequencing methods.

Features	Single-Cell RNA Sequencing	RNA Sequencing
Transcriptome analysis	Unique transcriptome expression of each of many diverse cell types	Average transcriptome expression of many cells
Heterogeneity of the population	Cell to cell variability	Cells from tissues considered to be homogeneous
Populations of cells	Identifying rare populations	-
Conditions of cells	Cells from one condition are generally captured and sequenced	Compares differentially expressed genes under multiple conditions
Statistical power	Increased (capturing thousands of cells in one condition)	-
Cells uniqueness	Revealing latent changes, new cell types, cells subpopulations	-
Generated data	Noisier, more variable data	Less background noise, less variable
Capture efficiency	Low	High

## References

[1] Xu J., Huang H., Wang S., Chen Y., Yin X., Zhang X., Zhang Y. (2019). Molecular profiling of TOX-deficient neoplastic cells in cutaneous T cell lymphoma. Arch. Dermatol. Res..

[2] Bastidas Torres A.N., Najidh S., Tensen C.P., Vermeer M.H. (2018). Molecular advances in cutaneous T-cell lymphoma. Semin. Cutan. Med. Surg..

[3] Cerroni L. (2018). Mycosis fungoides-clinical and histopathologic features, differential diagnosis, and treatment. Semin. Cutan. Med. Surg..

[4] Spicknall K.E. (2018). Sezary syndrome-clinical and histopathologic features, differential diagnosis, and treatment. Semin. Cutan. Med. Surg..

[5] Vakiti A., Padala S.A., Singh D. (2020). Sezary Syndrome. StatPearls.

[6] Agar N., Morris S., Russell-Jones R., Hawk J., Whittaker S. (2009). Case report of four patients with erythrodermic cutaneous T-cell lymphoma and severe photosensitivity mimicking chronic actinic dermatitis. Br. J. Dermatol..

[7] Jawed S.I., Myskowski P.L., Horwitz S., Moskowitz A., Querfeld C. (2014). Primary cutaneous T-cell lymphoma (mycosis fungoides and Sezary syndrome): Part I. Diagnosis: Clinical and histopathologic features and new molecular and biologic markers. J. Am. Acad. Dermatol..

[8] Saulite I., Hoetzenecker W., Weidinger S., Cozzio A., Guenova E., Wehkamp U. (2016). Sezary Syndrome and Atopic Dermatitis: Comparison of Immunological Aspects and Targets. Biom. Res. Int..

[9] Roelens M., Delord M., Ram-Wolff C., Marie-Cardine A., Alberdi A., Maki G., Homyrda L., Bensussan A., Bagot M., Toubert A. (2017). Circulating and skin-derived Sezary cells: Clonal but with phenotypic plasticity. Blood.

[10] Buus T.B., Willerslev-Olsen A., Fredholm S., Blumel E., Nastasi C., Gluud M., Hu T., Lindahl L.M., Iversen L., Fogh H. (2018). Single-cell heterogeneity in Sezary syndrome. Blood Adv..

[11] Gaydosik A.M., Tabib T., Geskin L.J., Bayan C.A., Conway J.F., Lafyatis R., Fuschiotti P. (2019). Single-Cell Lymphocyte Heterogeneity in Advanced Cutaneous T-cell Lymphoma Skin Tumors. Clin. Cancer Res..

[12] Borcherding N., Voigt A.P., Liu V., Link B.K., Zhang W., Jabbari A. (2019). Single-Cell Profiling of Cutaneous T-Cell Lymphoma Reveals Underlying Heterogeneity Associated with Disease Progression. Clin. Cancer Res..

[13] McGranahan N., Swanton C. (2017). Clonal Heterogeneity and Tumor Evolution: Past, Present, and the Future. Cell.

[14] Miyagaki T., Sugaya M. (2014). Immunological milieu in mycosis fungoides and Sezary syndrome. J. Dermatol..

[15] Stark R., Grzelak M., Hadfield J. (2019). RNA sequencing: The teenage years. Nat. Rev. Genet..

[16] Liu X., Ma Y., Yin K., Li W., Chen W., Zhang Y., Zhu C., Li T., Han B., Liu X. (2019). Long non-coding and coding RNA profiling using strand-specific RNA-seq in human hypertrophic cardiomyopathy. Sci. Data.

[17] Uphoff C.C., Pommerenke C., Denkmann S.A., Drexler H.G. (2019). Screening human cell lines for viral infections applying RNA-Seq data analysis. PLoS ONE.

[18] Picelli S. (2017). Single-cell RNA-sequencing: The future of genome biology is now. RNA Biol..

[19] Hwang B., Lee J.H., Bang D. (2018). Single-cell RNA sequencing technologies and bioinformatics pipelines. Exp. Mol. Med..

[20] Olsen T.K., Baryawno N. (2018). Introduction to Single-Cell RNA Sequencing. Curr. Protoc. Mol. Biol..

[21] Chen G., Ning B., Shi T. (2019). Single-Cell RNA-Seq Technologies and Related Computational Data Analysis. Front. Genet..

[22] Litvinov I.V., Tetzlaff M.T., Thibault P., Gangar P., Moreau L., Watters A.K., Netchiporouk E., Pehr K., Prieto V.G., Rahme E. (2017). Gene expression analysis in Cutaneous T-Cell Lymphomas (CTCL) highlights disease heterogeneity and potential diagnostic and prognostic indicators. Oncoimmunology.

[23] Morimura S., Sugaya M., Suga H., Miyagaki T., Ohmatsu H., Fujita H., Asano Y., Tada Y., Kadono T., Sato S. (2014). TOX expression in different subtypes of cutaneous lymphoma. Arch. Dermatol. Res..

[24] Yu X., Luo Y., Liu J., Liu Y., Sun Q. (2015). TOX acts an oncological role in mycosis fungoides. PLoS ONE.

[25] Van Kester M.S., Borg M.K., Zoutman W.H., Out-Luiting J.J., Jansen P.M., Dreef E.J., Vermeer M.H., van Doorn R., Willemze R., Tensen C.P. (2012). A meta-analysis of gene expression data identifies a molecular signature characteristic for tumor-stage mycosis fungoides. J. Investig. Dermatol..

[26] Kopp K.L., Ralfkiaer U., Gjerdrum L.M., Helvad R., Pedersen I.H., Litman T., Jonson L., Hagedorn P.H., Krejsgaard T., Gniadecki R. (2013). STAT5-mediated expression of oncogenic miR-155 in cutaneous T-cell lymphoma. Cell Cycle.

[27] Lefrancois P., Tetzlaff M.T., Moreau L., Watters A.K., Netchiporouk E., Provost N., Gilbert M., Ni X., Sasseville D., Duvic M. (2017). TruSeq-Based Gene Expression Analysis of Formalin-Fixed Paraffin-Embedded (FFPE) Cutaneous T-Cell Lymphoma Samples: Subgroup Analysis Results and Elucidation of Biases from FFPE Sample Processing on the TruSeq Platform. Front. Med..

[28] Prasad A., Rabionet R., Espinet B., Zapata L., Puiggros A., Melero C., Puig A., Sarria-Trujillo Y., Ossowski S., Garcia-Muret M.P. (2016). Identification of Gene Mutations and Fusion Genes in Patients with Sezary Syndrome. J. Investig. Dermatol..

[29] Izykowska K., Przybylski G.K., Gand C., Braun F.C., Grabarczyk P., Kuss A.W., Olek-Hrab K., Bastidas Torres A.N., Vermeer M.H., Zoutman W.H. (2017). Genetic rearrangements result in altered gene expression and novel fusion transcripts in Sezary syndrome. Oncotarget.

[30] Wang L., Ni X., Covington K.R., Yang B.Y., Shiu J., Zhang X., Xi L., Meng Q., Langridge T., Drummond J. (2015). Genomic profiling of Sezary syndrome identifies alterations of key T cell signaling and differentiation genes. Nat. Genet..

[31] Chase A., Ernst T., Fiebig A., Collins A., Grand F., Erben P., Reiter A., Schreiber S., Cross N.C. (2010). TFG, a target of chromosome translocations in lymphoma and soft tissue tumors, fuses to GPR128 in healthy individuals. Haematologica.

[32] Yoo H.Y., Kim P., Kim W.S., Lee S.H., Kim S., Kang S.Y., Jang H.Y., Lee J.E., Kim J., Kim S.J. (2016). Frequent CTLA4-CD28 gene fusion in diverse types of T-cell lymphoma. Haematologica.

[33] Sekulic A., Liang W.S., Tembe W., Izatt T., Kruglyak S., Kiefer J.A., Cuyugan L., Zismann V., Legendre C., Pittelkow M.R. (2015). Personalized treatment of Sezary syndrome by targeting a novel CTLA4:CD28 fusion. Mol. Genet. Genom. Med..

[34] O’Donnell J.S., Smyth M.J., Teng M.W.L. (2017). PD1 functions by inhibiting CD28-mediated co-stimulation. Clin. Transl. Immunol..

[35] Khodadoust M.S., Rook A.H., Porcu P., Foss F., Moskowitz A.J., Shustov A., Shanbhag S., Sokol L., Fling S.P., Ramchurren N. (2020). Pembrolizumab in Relapsed and Refractory Mycosis Fungoides and Sezary Syndrome: A Multicenter Phase II Study. J. Clin. Oncol..

[36] Suga H., Sugaya M., Miyagaki T., Kawaguchi M., Fujita H., Asano Y., Tada Y., Kadono T., Sato S. (2014). The role of IL-32 in cutaneous T-cell lymphoma. J. Investig. Dermatol..

[37] Fernandes J.C.R., Acuna S.M., Aoki J.I., Floeter-Winter L.M., Muxel S.M. (2019). Long Non-Coding RNAs in the Regulation of Gene Expression: Physiology and Disease. Noncoding RNA.

[38] Chi Y., Wang D., Wang J., Yu W., Yang J. (2019). Long Non-Coding RNA in the Pathogenesis of Cancers. Cells.

[39] DiStefano J.K. (2018). The Emerging Role of Long Noncoding RNAs in Human Disease. Methods Mol. Biol..

[40] Salamon I., Saccani Jotti G., Condorelli G. (2018). The long noncoding RNA landscape in cardiovascular disease: A brief update. Curr. Opin. Cardiol..

[41] Galamb O., Bartak B.K., Kalmar A., Nagy Z.B., Szigeti K.A., Tulassay Z., Igaz P., Molnar B. (2019). Diagnostic and prognostic potential of tissue and circulating long non-coding RNAs in colorectal tumors. World J. Gastroenterol..

[42] Xie Y., Zhang Y., Du L., Jiang X., Yan S., Duan W., Li J., Zhan Y., Wang L., Zhang S. (2018). Circulating long noncoding RNA act as potential novel biomarkers for diagnosis and prognosis of non-small cell lung cancer. Mol. Oncol..

[43] Matsui M., Corey D.R. (2017). Non-coding RNAs as drug targets. Nat. Rev. Drug Discov..

[44] Tripathi R., Chakraborty P., Varadwaj P.K. (2017). Unraveling long non-coding RNAs through analysis of high-throughput RNA-sequencing data. Noncoding RNA Res..

[45] Lee C.S., Ungewickell A., Bhaduri A., Qu K., Webster D.E., Armstrong R., Weng W.K., Aros C.J., Mah A., Chen R.O. (2012). Transcriptome sequencing in Sezary syndrome identifies Sezary cell and mycosis fungoides-associated lncRNAs and novel transcripts. Blood.

[46] Wong H.K., Mishra A., Hake T., Porcu P. (2011). Evolving insights in the pathogenesis and therapy of cutaneous T-cell lymphoma (mycosis fungoides and Sezary syndrome). Br. J. Haematol..

[47] Pomerantz R.G., Mirvish E.D., Erdos G., Falo L.D., Geskin L.J. (2010). Novel approach to gene expression profiling in Sezary syndrome. Br. J. Dermatol..

[48] Booken N., Gratchev A., Utikal J., Weiss C., Yu X., Qadoumi M., Schmuth M., Sepp N., Nashan D., Rass K. (2008). Sezary syndrome is a unique cutaneous T-cell lymphoma as identified by an expanded gene signature including diagnostic marker molecules CDO1 and DNM3. Leukemia.

[49] Kamstrup M.R., Gjerdrum L.M., Biskup E., Lauenborg B.T., Ralfkiaer E., Woetmann A., Odum N., Gniadecki R. (2010). Notch1 as a potential therapeutic target in cutaneous T-cell lymphoma. Blood.

[50] Di L., Srivastava S., Zhdanova O., Ding Y., Li Z., Wulff H., Lafaille M., Skolnik E.Y. (2010). Inhibition of the K+ channel KCa3.1 ameliorates T cell-mediated colitis. Proc. Natl. Acad. Sci. USA.

[51] Siwiec A., Majdan M. (2015). The role of the PD-1 protein in pathogenesis of autoimmune diseases, with particular consideration of rheumatoid arthritis and systemic lupus erythematosus. Postepy Hig. Med. Dosw..

[52] Loo D.T., Mather J.P. (2008). Antibody-based identification of cell surface antigens: Targets for cancer therapy. Curr. Opin. Pharmacol..

[53] Ohnuma K., Dang N.H., Morimoto C. (2008). Revisiting an old acquaintance: CD26 and its molecular mechanisms in T cell function. Trends Immunol..

[54] Murphy M., Fullen D., Carlson J.A. (2002). Low CD7 expression in benign and malignant cutaneous lymphocytic infiltrates: Experience with an antibody reactive with paraffin-embedded tissue. Am. J. Dermatopathol..

[55] Choi J., Goh G., Walradt T., Hong B.S., Bunick C.G., Chen K., Bjornson R.D., Maman Y., Wang T., Tordoff J. (2015). Genomic landscape of cutaneous T cell lymphoma. Nat. Genet..

[56] Da Silva Almeida A.C., Abate F., Khiabanian H., Martinez-Escala E., Guitart J., Tensen C.P., Vermeer M.H., Rabadan R., Ferrando A., Palomero T. (2015). The mutational landscape of cutaneous T cell lymphoma and Sezary syndrome. Nat. Genet..

[57] Izban K.F., Ergin M., Qin J.Z., Martinez R.L., Pooley R.J., Saeed S., Alkan S. (2000). Constitutive expression of NF-kappa B is a characteristic feature of mycosis fungoides: Implications for apoptosis resistance and pathogenesis. Hum. Pathol..

[58] Ungewickell A., Bhaduri A., Rios E., Reuter J., Lee C.S., Mah A., Zehnder A., Ohgami R., Kulkarni S., Armstrong R. (2015). Genomic analysis of mycosis fungoides and Sezary syndrome identifies recurrent alterations in TNFR2. Nat. Genet..

[59] Mirvish J.J., Pomerantz R.G., Falo L.D., Geskin L.J. (2013). Role of infectious agents in cutaneous T-cell lymphoma: Facts and controversies. Clin. Dermatol..

[60] Abrams J.T., Balin B.J., Vonderheid E.C. (2001). Association between Sezary T cell-activating factor, Chlamydia pneumoniae, and cutaneous T cell lymphoma. Ann. N. Y. Acad. Sci..

[61] Nedoszytko B., Wierzbicki P., Karenko L., Maciejewska-Radomska A., Stachewicz P., Zablotna M., Glen J., Vakeva L., Nowicki R.J., Sokolowska-Wojdylo M. (2018). Presence of Chlamydophila pneumoniae DNA in blood cells is a frequent event in patients with the late stage of primary cutaneous lymphomas and with atopic dermatitis. Postepy Dermatol. Alergol..

[62] Ponzoni M., Ferreri A.J., Mappa S., Pasini E., Govi S., Facchetti F., Fanoni D., Tucci A., Vino A., Doglioni C. (2011). Prevalence of Borrelia burgdorferi infection in a series of 98 primary cutaneous lymphomas. Oncologist.

[63] Tothova S.M., Bonin S., Trevisan G., Stanta G. (2006). Mycosis fungoides: Is it a Borrelia burgdorferi-associated disease?. Br. J. Cancer.

[64] Nguyen V., Huggins R.H., Lertsburapa T., Bauer K., Rademaker A., Gerami P., Guitart J. (2008). Cutaneous T-cell lymphoma and Staphylococcus aureus colonization. J. Am. Acad. Dermatol..

[65] Talpur R., Bassett R., Duvic M. (2008). Prevalence and treatment of Staphylococcus aureus colonization in patients with mycosis fungoides and Sezary syndrome. Br. J. Dermatol..

[66] Lindahl L.M., Willerslev-Olsen A., Gjerdrum L.M.R., Nielsen P.R., Blumel E., Rittig A.H., Celis P., Herpers B., Becker J.C., Stausbol-Gron B. (2019). Antibiotics inhibit tumor and disease activity in cutaneous T-cell lymphoma. Blood.

[67] Pancake B.A., Zucker-Franklin D., Coutavas E.E. (1995). The cutaneous T cell lymphoma, mycosis fungoides, is a human T cell lymphotropic virus-associated disease. A study of 50 patients. J. Clin. Investig..

[68] Zucker-Franklin D., Coutavas E.E., Rush M.G., Zouzias D.C. (1991). Detection of human T-lymphotropic virus-like particles in cultures of peripheral blood lymphocytes from patients with mycosis fungoides. Proc. Natl. Acad. Sci. USA.

[69] Zucker-Franklin D., Hooper W.C., Evatt B.L. (1992). Human lymphotropic retroviruses associated with mycosis fungoides: Evidence that human T-cell lymphotropic virus type II (HTLV-II) as well as HTLV-I may play a role in the disease. Blood.

[70] Bazarbachi A., Saal F., Laroche L., Flageul B., Peries J., de The H. (1993). HTLV-1 provirus and mycosis fungoides. Science.

[71] Bazarbachi A., Soriano V., Pawson R., Vallejo A., Moudgil T., Matutes E., Peries J., Molina A., de The H., Schulz T.F. (1997). Mycosis fungoides and Sezary syndrome are not associated with HTLV-I infection: An international study. Br. J. Haematol..

[72] Boni R., Davis-Daneshfar A., Burg G., Fuchs D., Wood G.S. (1996). No detection of HTLV-I proviral DNA in lesional skin biopsies from Swiss and German patients with cutaneous T-cell lymphoma. Br. J. Dermatol..

[73] Courgnaud V., Duthanh A., Guillot B., Sitbon M., Dereure O. (2009). Absence of HTLV-related sequences in skin lesions and peripheral blood of cutaneous T-cell lymphomas. J. Investig. Dermatol..

[74] Pawlaczyk M., Filas V., Sobieska M., Gozdzicka-Jozefiak A., Wiktorowicz K., Breborowicz J. (2005). No evidence of HTLV-I infection in patients with mycosis fungoides and Sezary syndrome. Neoplasma.

[75] Poiesz B., Dube D., Dube S., Love J., Papsidero L., Uner A., Hutchinson R. (2000). HTLV-II-associated cutaneous T-cell lymphoma in a patient with HIV-1 infection. N. Engl. J. Med..

[76] Bachelez H., Hadida F., Gorochov G. (1996). Massive infiltration of the skin by HIV-specific cytotoxic CD8+ T cells. N. Engl. J. Med..

[77] Wilkins K., Turner R., Dolev J.C., LeBoit P.E., Berger T.G., Maurer T.A. (2006). Cutaneous malignancy and human immunodeficiency virus disease. J. Am. Acad. Dermatol..

[78] Gahongayire F. (2007). Mycosis fungoides and Sezary syndrome against a human immunodeficiency virus-positive background: Case report. Int. J. Dermatol..

[79] Haverkos B.M., Gru A.A., Geyer S.M., Bingman A.K., Hemminger J.A., Mishra A., Wong H.K., Pancholi P., Freud A.G., Caligiuri M.A. (2016). Increased Levels of Plasma Epstein Barr Virus DNA Identify a Poor-Risk Subset of Patients with Advanced Stage Cutaneous T-Cell Lymphoma. Clin. Lymphoma Myeloma Leuk..

[80] Novelli M., Merlino C., Ponti R., Bergallo M., Quaglino P., Cambieri I., Comessatti A., Sidoti F., Costa C., Corino D. (2009). Epstein-Barr virus in cutaneous T-cell lymphomas: Evaluation of the viral presence and significance in skin and peripheral blood. J. Investig. Dermatol..

[81] Park S., Lee D.Y., Kim W.S., Ko Y.H. (2010). Primary cutaneous Epstein-Barr virus-associated T-cell lymphoproliferative disorder-2 cases with unusual, prolonged clinical course. Am. J. Dermatopathol..

[82] Tournadre A., D’Incan M., Dubost J.J., Franck F., Dechelotte P., Souteyrand P., Soubrier M. (2001). Cutaneous lymphoma associated with Epstein-Barr virus infection in 2 patients treated with methotrexate. Mayo Clin. Proc..

[83] Brice S.L., Jester J.D., Friednash M., Golitz L.E., Leahy M.A., Stockert S.S., Weston W.L. (1993). Examination of cutaneous T-cell lymphoma for human herpesviruses by using the polymerase chain reaction. J. Cutan. Pathol..

[84] Erkek E., Senturk N., Dincer I., Olut A.I., Kocagoz T., Bukulmez G., Sahin S. (2002). Identification of herpes simplex virus DNA and lack of human herpesvirus-8 DNA in mycosis fungoides. Acta Derm. Venereol..

[85] Kreuter A., Bischoff S., Skrygan M., Wieland U., Brockmeyer N.H., Stucker M., Altmeyer P., Gambichler T. (2008). High association of human herpesvirus 8 in large-plaque parapsoriasis and mycosis fungoides. Arch. Dermatol..

[86] Ponti R., Bergallo M., Costa C., Quaglino P., Fierro M.T., Comessatti A., Stroppiana E., Sidoti F., Merlino C., Novelli M. (2008). Human herpesvirus 7 detection by quantitative real time polymerase chain reaction in primary cutaneous T-cell lymphomas and healthy subjects: Lack of a pathogenic role. Br. J. Dermatol..

[87] Bergallo M., Dapra V., Fava P., Ponti R., Calvi C., Montanari P., Novelli M., Quaglino P., Galliano I., Fierro M.T. (2018). DNA from Human Polyomaviruses, MWPyV, HPyV6, HPyV7, HPyV9 and HPyV12 in Cutaneous T-cell Lymphomas. Anticancer Res..

[88] Kreuter A., Silling S., Dewan M., Stucker M., Wieland U. (2011). Evaluation of 4 recently discovered human polyomaviruses in primary cutaneous B-cell and T-cell lymphoma. Arch. Dermatol..

[89] Miertusova S., Bonin S., Trevisan G., Stanta G. (2004). Mycosis fungoides is not associated with hepatitis C virus infection. Br. J. Dermatol..

[90] Netchiporouk E., Gantchev J., Tsang M., Thibault P., Watters A.K., Hughes J.M., Ghazawi F.M., Woetmann A., Odum N., Sasseville D. (2017). Analysis of CTCL cell lines reveals important differences between mycosis fungoides/Sezary syndrome vs. HTLV-1(+) leukemic cell lines. Oncotarget.

[91] Schmidt A.N., Robbins J.B., Greer J.P., Zic J.A. (2006). Conjugal transformed mycosis fungoides: The unknown role of viral infection and environmental exposures in the development of cutaneous T-cell lymphoma. J. Am. Acad. Dermatol..

[92] Weder P., Anliker M., Itin P., Bargetzi M. (2004). Familial cutaneous mycosis fungoides: Successful treatment with a combination of gemcitabine and alemtuzumab. Dermatology.

[93] Lozano A., Duvic M. (2007). Cutaneous T-cell lymphoma in non-blood-related family members: Report of an additional case. J. Am. Acad. Dermatol..

[94] Andrews J.M., Schmidt J.A., Carson K.R., Musiek A.C., Mehta-Shah N., Payton J.E. (2019). Novel cell adhesion/migration pathways are predictive markers of HDAC inhibitor resistance in cutaneous T cell lymphoma. EBioMedicine.

